# Population structure of giraffes is affected by management in the Great Rift Valley, Kenya

**DOI:** 10.1371/journal.pone.0189678

**Published:** 2018-01-03

**Authors:** Zoe Muller

**Affiliations:** 1 School of Biological Sciences, University of Bristol, Bristol, United Kingdom; 2 Giraffe Research and Conservation Trust, Nairobi, Kenya; University of Tasmania, AUSTRALIA

## Abstract

Giraffe populations in East Africa have declined in the past thirty years yet there has been limited research on this species. This study had four objectives: i) to provide a baseline population assessment for the two largest populations of Rothschild’s giraffes in Kenya, ii) to assess whether there are differences in population structure between the two enclosed populations, iii) to assess the potential and possible implications of different management practices on enclosed giraffe populations to inform future decision-making, and iv) to add to the availability of information available about giraffes in the wild. I used individual identification to assess the size and structure of the two populations; in Soysambu Conservancy between May 2010 and January 2011, I identified 77 giraffes; in Lake Nakuru National Park between May 2011 and January 2012, I identified 89. Population structure differed significantly between the two sites; Soysambu Conservancy contained a high percentage of juveniles (34%) and subadults (29%) compared to Lake Nakuru NP, which contained fewer juveniles (5%) and subadults (15%). During the time of this study Soysambu Conservancy contained no lions while Lake Nakuru NP contained a high density of lions (30 lions per 100km^2^). Lions are the main predator of giraffes, and preferential predation on juvenile giraffes has previously been identified in Lake Nakuru NP. My results suggest that high lion density in Lake Nakuru NP may have influenced the structure of the giraffe population by removing juveniles and, consequently, may affect future population growth. I suggest that wildlife managers consider lion densities alongside breeding plans for Endangered species, since the presence of lions appears to influence the population structure of giraffes in enclosed habitats.

## Introduction

Information about the size and distribution of giraffe populations is scarce and often outdated. A species-wide assessment by the IUCN Giraffe and Okapi Specialist Group was completed in 2016 [1], but information on subspecies population sizes and distributions is still incomplete. Most population size data for giraffes is gleaned from general wildlife counts carried out across large areas [2–5], which give incomplete indications of abundance and distribution. Currently, information about giraffe population structure or demography is limited and comes from a few studies of single populations. Despite their scarcity, such data are becoming increasingly important for understanding population changes, ecosystem dynamics and for the purposes of population monitoring, management and species conservation [1]. The 2016 IUCN *Giraffa camelopardalis* assessment and increasing evidence of steeply declining populations [1] has highlighted a lack of knowledge about this species and an urgent need for research into conservation and management.

In this study I used individual identification to study the two of the largest populations of Rothschild’s giraffes in Kenya; those within Lake Nakuru National Park (LNNP) and Soysambu Conservancy (SC). LNNP has a high density of lions and is Kenya’s most visited National Park [6, 7]. Consequently, the giraffe population in LNNP is exposed to a high risk of predation and frequent disturbance from high levels of tourist activity and vehicular traffic. In the adjacent SC at the time of this study there were no lions and minimal tourism activities, so giraffes in this site were exposed to low, or no, risk of predation, and minimal disturbance from limited vehicular activity. Reports have previously suggested that lions in LNNP preferentially prey on giraffe calves [8] but it is unclear what the consequences of this are, if any. If predation risk by lions has a significant impact upon giraffe calf survival, then I expect that LNNP will have significantly fewer calves than the population in SC.

This study had the following aims: i) to provide a baseline population assessment for each population, ii) to compare age class structures, group composition and sex ratio between sites, iii) to analyse the patterns of giraffe population size change over time in LNNP alongside lion densities, iv) to add to the availability of information about giraffes in the wild. All four objectives were achieved, and I provide discussion and commentary about how the results can be applied to conservation practice.

## Methods

### Study sites

**Fig 1:**
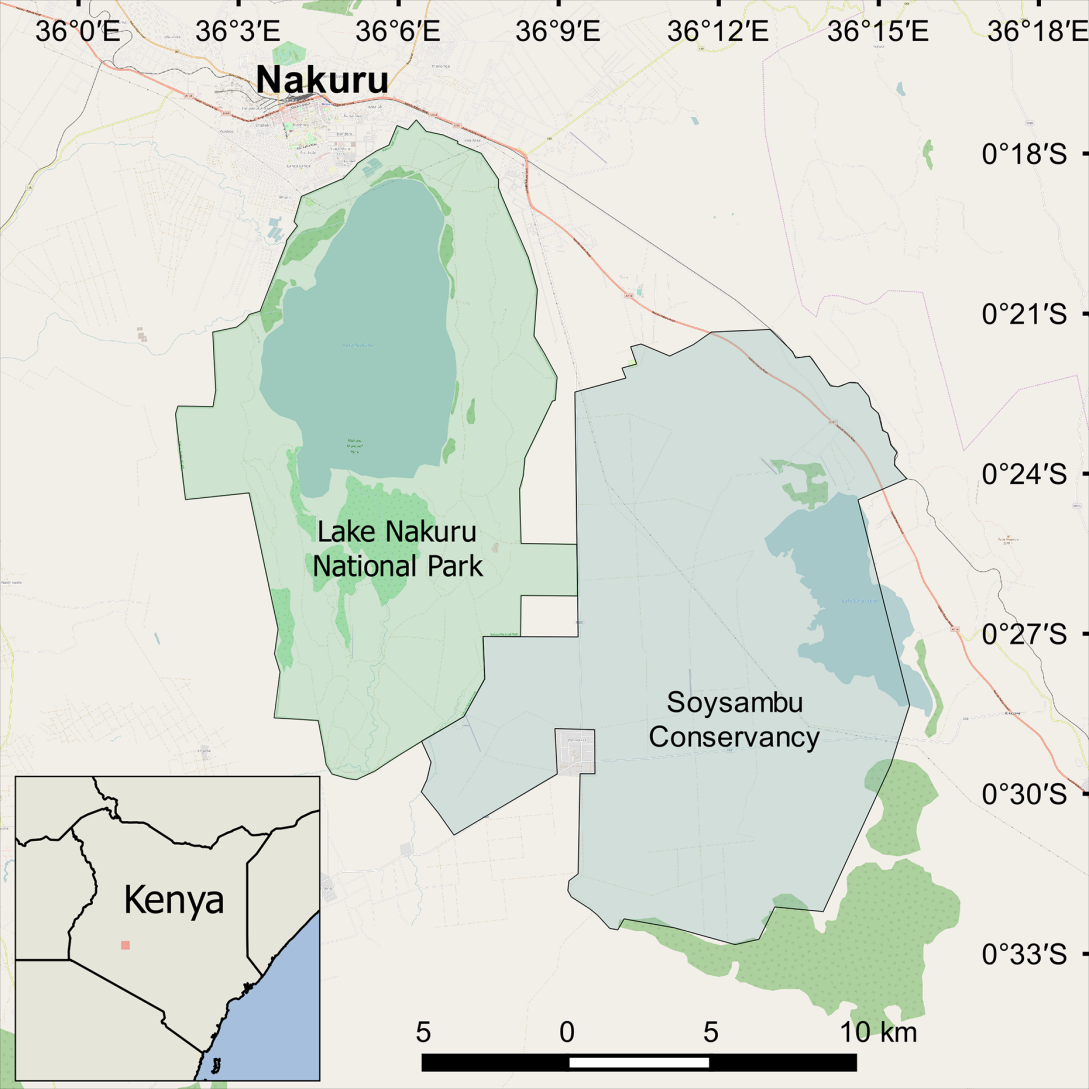
Location of the study sites in the Rift valley region of Kenya.

The study was conducted in the Great Rift Valley region of Kenya between May 2010 and January 2012. This region is classified as dry sub-humid to semi-arid with a mean annual rainfall of 920 mm/yr [9]. The giraffe population here is fragmented and only exists within confined conservation areas [1, 8]. I studied two discrete populations of giraffes; one confined within Soysambu Conservancy (SC) and one confined within Lake Nakuru National Park (LNNP). Both areas are enclosed and protected wildlife conservation areas situated adjacent to one another and part of the same biome, but separated by an electrified game fence across which there is no movement of large mammals (Fig 1). The habitat in both study sites consists of large patches of *Acacia* species and mixed woodland interspersed with open savannah grassland. SC is a privately-owned wildlife conservancy, 190km^2^ in size surrounding Lake Elementeita (00°46'S, 036°23'E; 1670m asl). LNNP is a National Park, 188km^2^ in size surrounding Lake Nakuru (0°22’S 36°05’E; 1759m asl).

At the time of this study SC was free of lions, and had been for several decades. Inside LNNP there were 56 lions [10], i.e. a high density of 0.3 individuals per km^2^. In areas with no exposure to hunting, typical lion densities are between 0.08 and 0.14 individuals per km^2^ [11, 12]. Lions are the only predator to pose a significant threat to giraffes [13–17] and the preferential preying of lions upon giraffes has been identified as a potential problem in LNNP [8].

### Data collection

To satisfy objectives i) and ii) I monitored the giraffe population in each study site for a period of nine continuous months (in SC from May 2010 to January 2011, in LNNP from May 2011 to January 2012). It was not possible to conduct concurrent monitoring so I time-matched data collection periods between consecutive years, to reduce any possible seasonal effects as far as possible. All data were collected by driving a 4x4 vehicle along transects to search for giraffes opportunistically. Each study site was split into four blocks (NE, SE, SW, NW); each day the order that the blocks were searched was randomised, and the road network within each block was driven in a random order (direction and road chosen). All roads in all blocks were driven each day so that all areas were searched. Data were collected between sunrise at 06:30 and sunset at 18:30 (UTC + 3h Standard Time). Upon sighting an individual or group of giraffes, I stopped the vehicle at an appropriate distance so as not to disturb the animals (typically 100 to 500 metres) and group composition data were recorded. I defined a group of giraffes as ‘all individuals being within 1km of each other and engaged in generally similar behaviour’ [18–23]. There were no instances where an individual could be considered at the threshold of this definition.

Upon stopping the vehicle I took a photograph of each individual in the group from the furthermost left animal to the furthermost right animal, to create a digital record of the observation. I took further photographs of group members opportunistically as they moved or turned around, to provide further records of group members and to verify identifications. I used a Nikon D90 digital SLR camera with a 50-500mm Sigma lens. I initially made all identifications in the field, but later checked these for accuracy using the digital images. I examined every digital photograph to ensure I had allocated the correct identification, age class and sex to each animal. Group type was categorised as one of six types: lone male, lone female, mixed sex, females, males, females and juveniles. There were no instances where only males and juveniles were found in a group together.

### Individual identification

All giraffes in each study site were individually recognised and classified by sex and age class. Each giraffe was identified using its unique coat pattern [22, 24, 25] (S1 Fig) and was allocated a unique identifying code (S2 Fig). An identification (ID) file was created for each giraffe including photos of its left and right sides (S3 Fig). Sex was determined by observing general physical characteristics (S4 Fig) and the shape of the giraffe’s skull; the skulls of female giraffes are narrower in angle when viewed side-on than those of males [26–28] (females S5 Fig; males S6 Fig). Accurate age classification of wild giraffes is difficult without an individual’s birth data so age classes are widely used in field studies [13, 19, 22, 24, 29–31]. Age classes were categorised as juvenile (<12 months), subadult (12 months to < 4 years), or adult (4+ years). Mature adult males with dark coats and skull nodules, estimated to be 9+ years [25, 32] were classified as big bulls [29, 31] (S7 and S8 Figs). For comparison with recent literature which uses an A/B/C system of classifying males [29, 33], in this study ‘big bulls’ are equivalent to category A males, and ‘adult males’ are equivalent to category B and C males combined. Juveniles were not sexed because the presence of the umbilicus and small body size made accurate sex-identification challenging (S9 Fig).

Individuals were allocated unique identifying codes on the first instance that I reliably observed both the left and right sides in one observation. If I failed to photograph both sides of an animal at the same observation, then that individual was not allocated an identifying code until a time when both sides could be photographed in the same observation to ensure accuracy. I considered the ID catalogue complete when no new individuals had been identified for a period of two consecutive weeks. Following completion of the ID catalogue in each site no new individuals were found (except for juveniles born during the study period).

### Population size and survey effort summaries

Total population size and structure in each study site was established by examining the completed individual identification records, and totalling the number of giraffes identified in each age and sex class. I used SOCPROG 2.7 [34] to generate summary statistics to describe the min., max. and mean times each giraffe was seen, and the mean percentage of the population observed on each survey. I used a t-test to check for bias in the volume of observations between study sites, and a chi-square test to check for differences in population structure between sites, both in R [35].

### Approvals and permissions

The methods used in this study were observational and non-invasive. Ethical approval was granted by the University of Bristol Ethical Review Group, and the study was carried out under permit number NCST/RRI/12/1/MAS/08/5 with the authorisation of the Kenya Wildlife Service and the Kenya National Council for Science and Technology.

## Results

### Volume of encounters

In SC 1,442 sightings of individual giraffes were recorded in 298 groups with a mean of 23 giraffes (32% of the population) sighted each survey (i.e. each day). Each individual giraffe was sighted between 1 and 36 times (mean = 16, *SD* = 8). Males were sighted a mean of 15 times (*SD* = 5.13) and females a mean of 23 times (*SD* = 7.91). In LNNP 1,438 sightings of individual giraffes were recorded in 293 groups with a mean of 25 giraffes (28% of the population) sighted per survey. Each individual giraffe was observed between 1 and 26 times (mean = 16, *SD* = 6). Males were sighted a mean of 16 times (*SD* = 5.61) and females a mean of 17 times (*SD* = 4.75). There was no significant difference in the number of times each individual was observed between study sites (*t*(163) = 0.11, *p* = 0.913). S1 Table lists the individuals present in each study site with age and sex class information, and number of times sighted. Individuals sighted fewer than five times either died at the start of the study period or were born towards the end of the study period.

### Population size and structure

**Fig 2:**
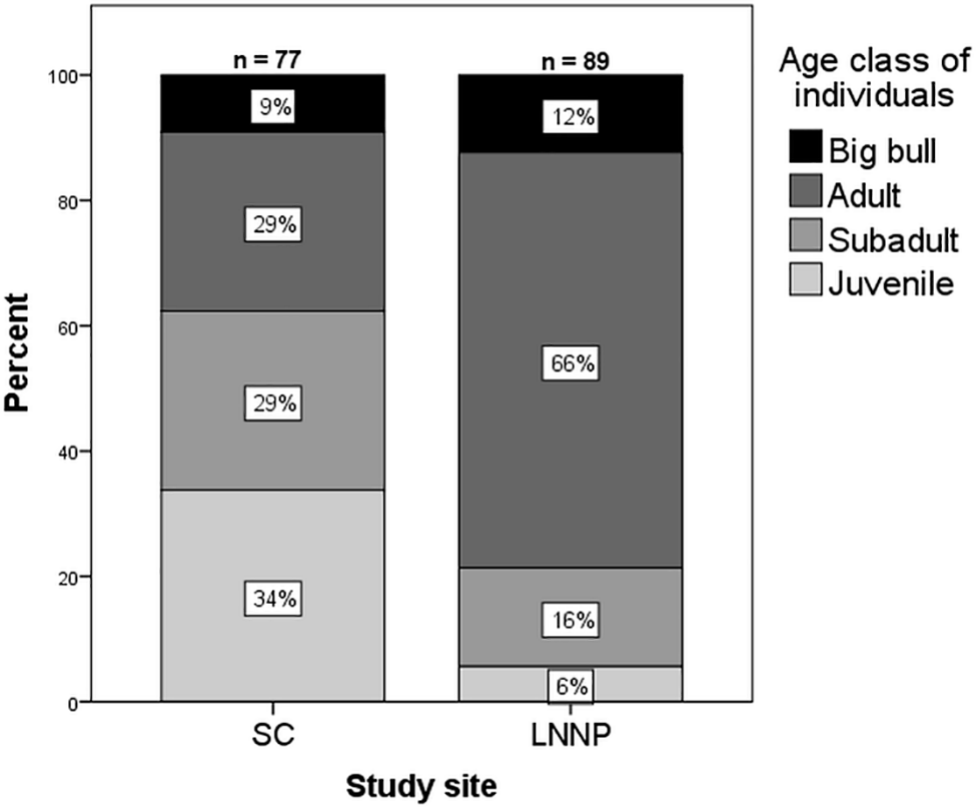
Giraffe age class structure of each study population.

In SC I identified 77 giraffes; in LNNP I identified 89 giraffes. The proportions of each age class between the two populations were significantly different (χ^2^ = 34.225, df = 5, p < 0.0001) (Fig 2). Of the giraffes which could be sexed (i.e. those which were not juveniles: SC, *n* = 51; LNNP, *n* = 84), there were 51% females and 49% males in SC and 52% females and 48% males in LNNP.

### Group type frequency

**Fig 3:**
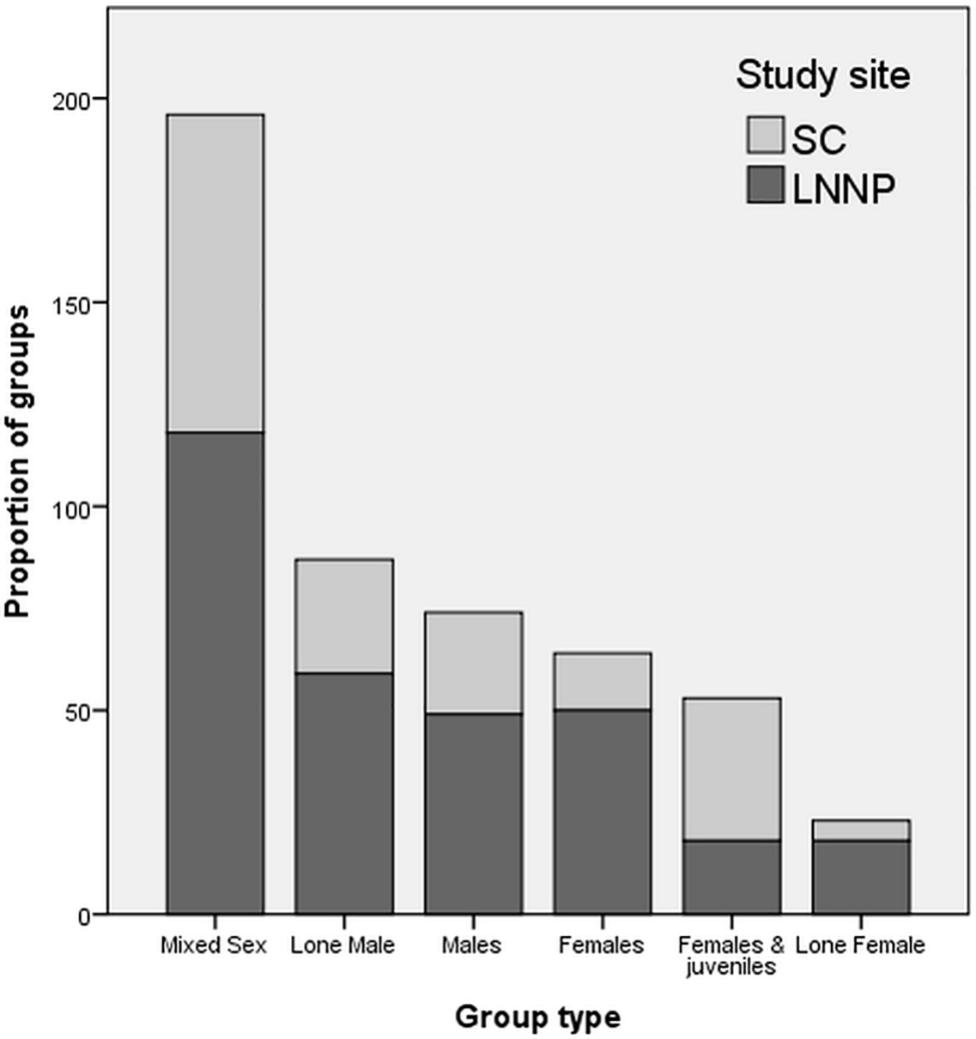
Frequency of different giraffe group types observed in each study site.

The most frequently observed group type was ‘mixed sex’ in both study sites, followed by ‘females and juveniles’ in SC, and ‘lone males’ in LNNP. Group type ‘lone female’ was the least observed in SC, while ‘lone female’ and ‘females and juveniles’ were the least observed group types in LNNP (Fig 3).

## Discussion

### Population size

Population figures for Rothschild’s giraffe in Kenya are scarce; the only population counts available for giraffes in Lake Nakuru National Park are 153 individuals in 1994 and 62 individuals in 2002 [36], implying that the population crashed by 91 individuals (59%) in an eight-year period. This crash is thought to have occurred due to a combination of factors including inbreeding depression, over-browsing of *Acacia* species, the heavy predation of giraffe calves by lions, and climatic effects [8]. My results suggest that the population within LNNP has grown by 27 individuals (44%) in the nine years since the last published survey in 2002. It has been suggested that LNNP is able to support 150 giraffes [8], so this growth may be the natural effects of a population recovering from a previous period of challenging conditions. The population growth could also be a result of a decrease in lion density; in 2002 there were an estimated 65 lions inside LNNP [36] compared to the 56 individuals counted in 2011 [10] (i.e. 35 lions/100km^2^ in 2002 vs. 30 lions/km^2^ in 2011). This represents a 14% decrease in lions during the period 2002 to 2011 and may be a factor contributing to the 44% growth of the giraffe population during that same period. Although it is impossible to determine the exact causes of the population growth, this study provides a possible and preliminary indication of how a decrease in lion density may contribute to an apparent recovery in the co-resident giraffe population. My observation of 77 giraffes within SC provide the first published population figure for the area, which can be used as a baseline for ongoing population monitoring.

### Population structure

The sex and age structure of the two populations differed significantly between the two sites; LNNP had a smaller proportion of juveniles and subadults, and a larger proportion of adults compared to SC (Fig 2). Given previous suggestions that lion predation contributed to the population decline observed between 1994 and 2002 [8] and previous reports of lions in LNNP preferentially preying on juvenile giraffes [36], it is likely that the observed age structure in LNNP is a result of lion predation removing juveniles from the population. Climatic factors or the amount of suitable diet available may also have influenced or restricted growth rates. Previous studies have shown that giraffes utilise forage resources differently according to age and sex class [37–39]. However, it would be expected that any climatic factors affect all age classes of giraffes similarly, and should not create the unequally distributed age structure observed in this study. Lion predation is the main cause of death for giraffe calves [15]; they are a preferred target for lions and rarely survive an attack [14]. My observation that LNNP contains so few juveniles appears to support this. The small number of subadults in LNNP (*n* = 14, 15% of total population vs. *n* = 22, 29% in SC) could indicate that the risk of lion predation is increasing, since previously enough juveniles have survived (i.e. 14) to reach subadulthood, while only 5 juveniles were alive during my observations. Future surveys may find fewer subadults as juveniles fail to reach their next life stage.

The sex structure of the population was within the typical range reported by previous studies [21] and the ratio of adult males to adult females was similar between the two study sites. This may suggest that lions do not discriminate between giraffes by sex when attacking. If juveniles are a preferred target for lions in LNNP [8, 36] then both male and female calves are equally as likely to be predated upon, leading to a 50–50 sex split in those which survive to be subadults and adults. In the Serengeti the prevalence of lion claw marks on giraffes were significantly higher in females than in males, but the authors suggested this was likely to reflect calf defence rather than a preference for attacking females, or that females engage in riskier behaviour [14].

### Group type frequency

Frequency of different group types of giraffes appears to vary between studies; with the most frequent group type having been reported to be males [31, 40] or mixed-sex groups [19, 21]. Such variation is likely to be due to seasonal effects and differing environmental conditions between studies. Group types of ‘mixed sex’ were the most frequently observed in both study sites and typically comprised adults, subadults and juveniles. ‘Females and young’ was the second most frequent group type observed in SC, but was one of the least observed group types in LNNP, which is likely to be an artefact of the different number of juveniles between each site. Group type ‘lone male’ was the second most frequently observed group type in LNNP, and third most in SC, which is unsurprising as adult males are known to spend more time alone, regardless of predation risk [15, 20, 21, 40–42]. The least frequently observed group type in both sites was ‘lone female’; the proportion of lone female groups was smallest in SC and is likely to be a reflection of limited opportunities to be alone, due to the high number of juveniles present.

### Suggestions for conservation

Giraffes are classified as Vulnerable on the IUCN Red List of Threatened Species [1] and populations in East Africa are generally in decline. Declines in the Lake Nakuru population between 1995 (153 individuals) and 2002 (63 individuals) have been attributed to climatic factors and a failure to recruit young into the population during those years [8] however this study demonstrates that the recruitment of young is an ongoing problem, which may be linked to high lion densities. In areas where the main objective is to encourage the population growth and range expansion of giraffes, I suggest that the presence of lions should be limited as far as possible. Future translocations intending to establish new populations of giraffes should select new areas of occupancy known to contain small densities of lions.

## Conclusion

This study demonstrates how wildlife management practices can affect the population structure and population growth potential of giraffes, and careful monitoring of the lion population in LNNP is necessary to further understand the relationship between lion density and giraffe population size in this area.

The four objectives of this study were achieved: i) I have provided a baseline population assessment for the two largest populations of Rothschild’s giraffes in Kenya for 2010–2011, which can be used for future monitoring; ii) I uncovered important structural differences between the two populations, and showed how individual identification can provide robust assessments of small populations of threatened species, iii) I propose that high lion densities may have an impact on giraffe population growth potential in enclosed habitats, and iv) this manuscript adds to the amount of information available about giraffes in the wild, and highlights the need for further research to contribute even basic information about this understudied species.

## Supporting information

S1 Fig Examples of the distinctive features of a giraffe’s coat pattern used for individual identification. (TIF)

S2 Fig Example of a unique ID code for the eighth male identified in Lake Nakuru National Park. (TIF)

S3 Fig Example of an individual giraffe identification file showing both sides of the animal. (TIF)

S4 Fig Differences in physical characteristics between female and male giraffes, allowing for sex identification in the field. (TIF)

S5 Fig Photographs of eight different female Rothschild’s giraffe to show general female head shape. Note the slim head, small backward-sloping ossicones and minimal protrusion on the front of the skull.
(TIF)

S6 Fig Photographs of eight different male Rothschild’s giraffe to show general male head shape. Note, in comparison to females, a broader head shape, wider angle from the muzzle to back of the skull, prominent protrusion on the front of the skull and large, upright ossicones.
(TIF)

S7 Fig Four different males, classified in this study as big bulls. Note the calcium deposits on their skulls, dark colour and distinct appearance.
(TIF)

S8 Fig A big bull (left) standing next to an adult male (right). Note the big bull’s dark coat colour and generally larger body size in relation to the adult male.
(TIF)

S9 Fig Two different juvenile giraffes at two weeks old with umbilicus still attached. Note the physical characteristics make it impossible to reliably determine sex through observation at this young age.
(TIF)

S1 Table List of individual giraffes within each study site with sex and age class characteristics defined, and the number of times they were observed during the study period listed. Individuals which were observed < 5 times either died towards the beginning of the study period, or were born towards the end.
(DOCX)
