# Supplementary figures and images for: Population structure of giraffes is affected by management in the Great Rift Valley, Kenya
Source: PLoS One. 2018 Jan 3;13(1):e0189678. doi: 10.1371/journal.pone.0189678 (PMC5751992; doi:10.1371/journal.pone.0189678)

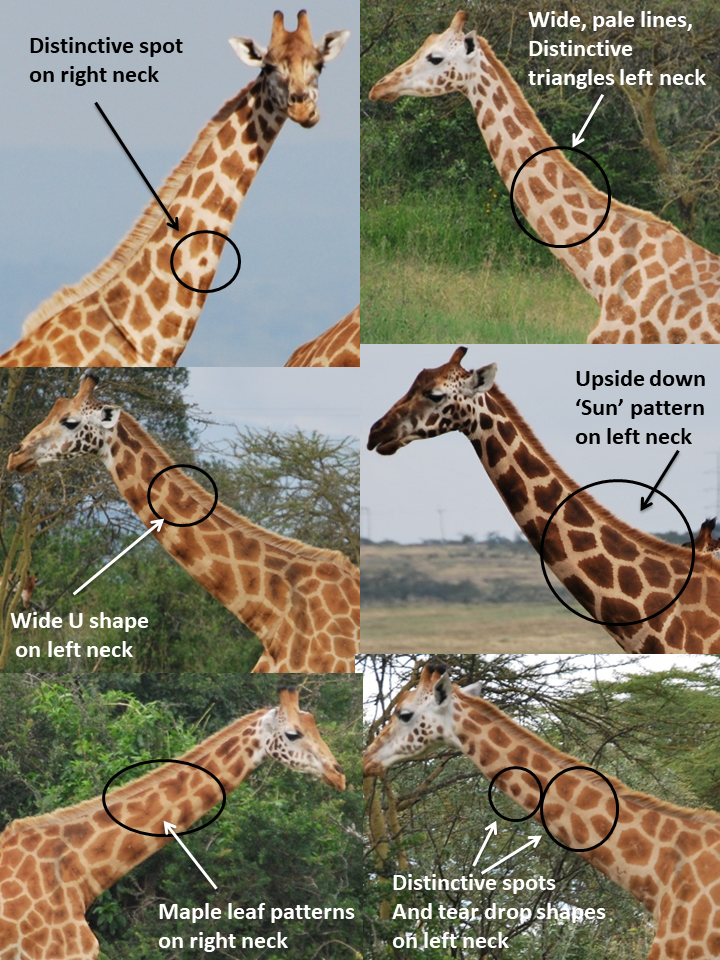

Supplement: S1 Fig — (TIF) [file pone.0189678.s001.tif]

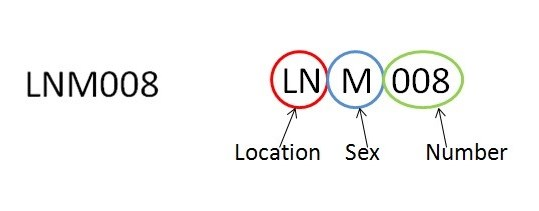

Supplement: S2 Fig — (TIF) [file pone.0189678.s002.tif]

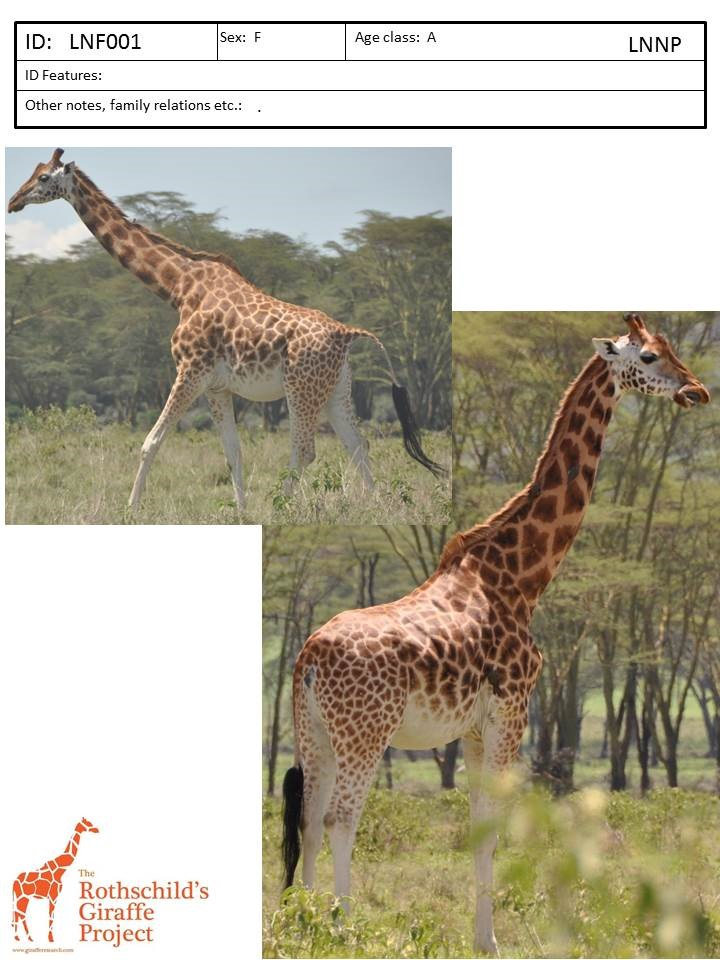

Supplement: S3 Fig — (TIF) [file pone.0189678.s003.tif]

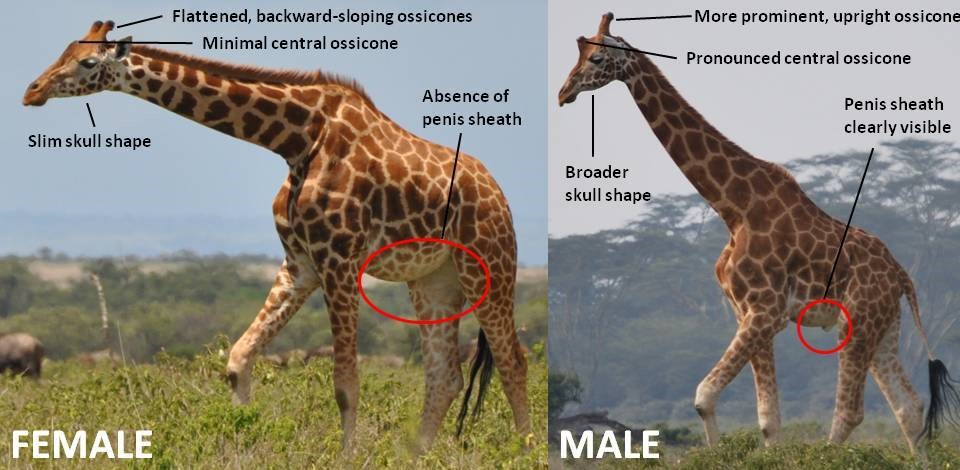

Supplement: S4 Fig — (TIF) [file pone.0189678.s004.tif]

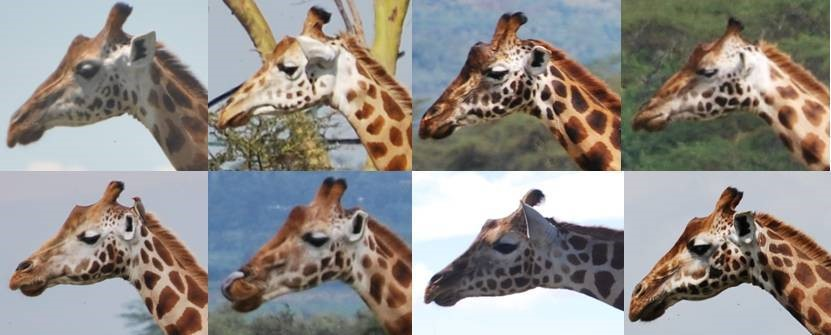

Supplement: S5 Fig — Note the slim head, small backward-sloping ossicones and minimal protrusion on the front of the skull. (TIF) [file pone.0189678.s005.tif]

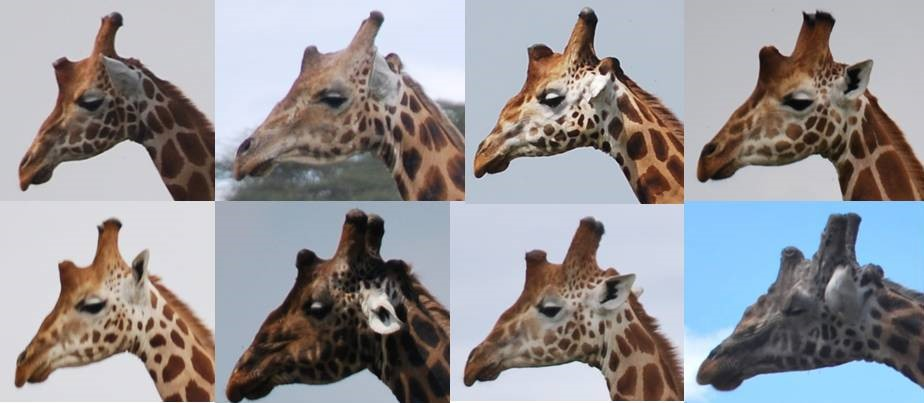

Supplement: S6 Fig — Note, in comparison to females, a broader head shape, wider angle from the muzzle to back of the skull, prominent protrusion on the front of the skull and large, upright ossicones. (TIF) [file pone.0189678.s006.tif]

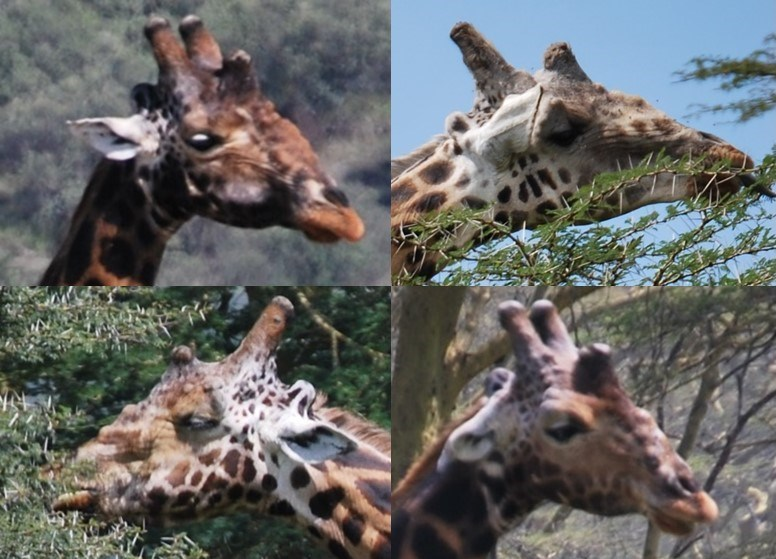

Supplement: S7 Fig — Note the calcium deposits on their skulls, dark colour and distinct appearance. (TIF) [file pone.0189678.s007.tif]

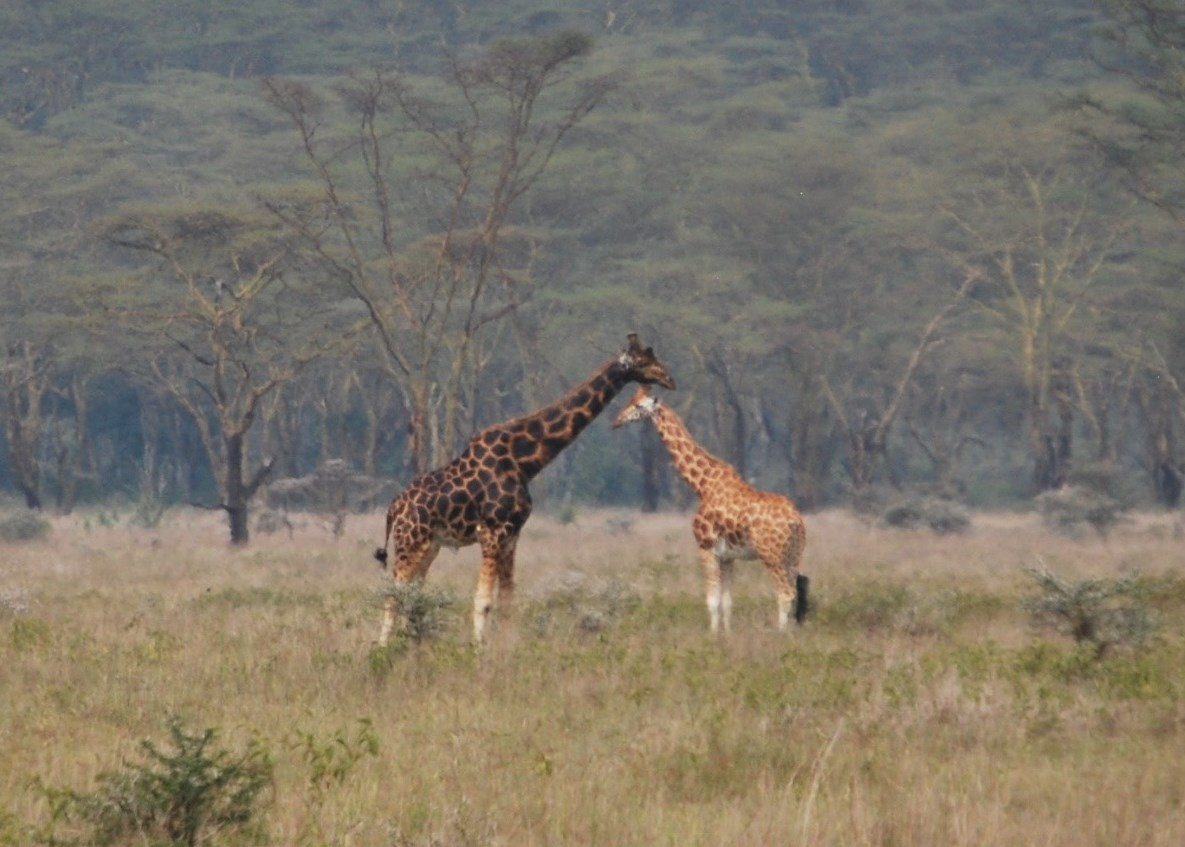

Supplement: S8 Fig — Note the big bull’s dark coat colour and generally larger body size in relation to the adult male. (TIF) [file pone.0189678.s008.tif]

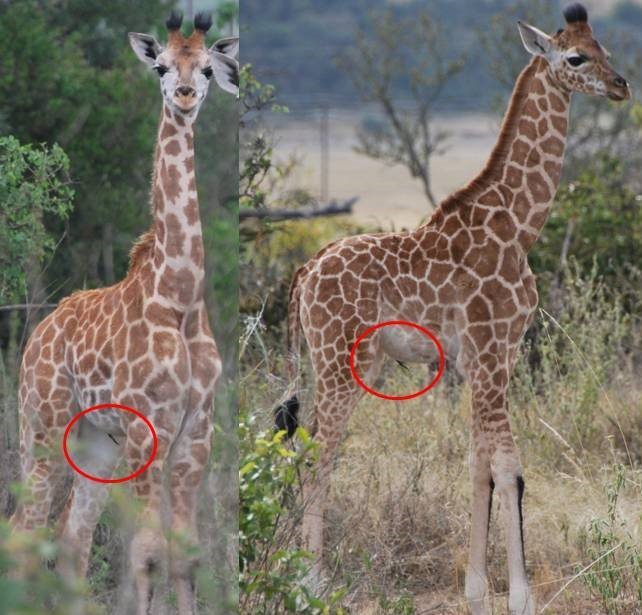

Supplement: S9 Fig — Note the physical characteristics make it impossible to reliably determine sex through observation at this young age. (TIF) [file pone.0189678.s009.tif]
