# Supplementary material for: Population structure of giraffes is affected by management in the Great Rift Valley, Kenya
Source: PLoS One. 2018 Jan 3;13(1):e0189678. doi: 10.1371/journal.pone.0189678 (PMC5751992; doi:10.1371/journal.pone.0189678)
Supplement: S1 Table — Individuals which were observed < 5 times either died towards the beginning of the study period, or were born towards the end. (DOCX) [file pone.0189678.s010.docx]

| **Study site** | **Giraffe ID** | **Sex** | **Age Class** | **No. of times observed during study period** |
| --- | --- | --- | --- | --- |
| SC | SCM001 | Male | Adult | 17 |
| SC | SCM002 | Male | Adult | 15 |
| SC | SCM003 | Male | Subadult | 12 |
| SC | SCM004 | Male | Big bull | 17 |
| SC | SCM005 | Male | Big bull | 16 |
| SC | SCM006 | Male | Big bull | 10 |
| SC | SCM007 | Male | Subadult | 16 |
| SC | SCM008 | Male | Subadult | 23 |
| SC | SCM009 | Male | Subadult | 20 |
| SC | SCM010 | Male | Subadult | 15 |
| SC | SCM011 | Male | Big bull | 9 |
| SC | SCM012 | Male | Adult | 17 |
| SC | SCM013 | Male | Adult | 20 |
| SC | SCM014 | Male | Adult | 10 |
| SC | SCM015 | Male | Adult | 15 |
| SC | SCM016 | Male | Adult | 13 |
| SC | SCM017 | Male | Adult | 12 |
| SC | SCM018 | Male | Adult | 11 |
| SC | SCM019 | Male | Big bull | 24 |
| SC | SCM020 | Male | Subadult | 16 |
| SC | SCM021 | Male | Big bull | 11 |
| SC | SCM022 | Male | Subadult | 1 |
| SC | SCM023 | Male | Big bull | 21 |
| SC | SCM024 | Male | Adult | 22 |
| SC | SCM025 | Male | Big bull | 17 |
| SC | SCM026 | Male | Subadult | 10 |
| SC | SCF001 | Female | Adult | 24 |
| SC | SCF002 | Female | Adult | 25 |
| SC | SCF003 | Female | Subadult | 30 |
| SC | SCF004 | Female | Adult | 24 |
| SC | SCF005 | Female | Adult | 24 |
| SC | SCF006 | Female | Adult | 30 |
| SC | SCF007 | Female | Adult | 33 |
| SC | SCF008 | Female | Adult | 23 |
| SC | SCF009 | Female | Adult | 20 |
| SC | SCF010 | Female | Adult | 25 |
| SC | SCF011 | Female | Subadult | 22 |
| SC | SCF012 | Female | Adult | 4 |
| SC | SCF013 | Female | Adult | 34 |
| SC | SCF014 | Female | Adult | 30 |
| SC | SCF015 | Female | Adult | 19 |
| SC | SCF016 | Female | Adult | 22 |
| SC | SCF017 | Female | Subadult | 23 |
| SC | SCF018 | Female | Adult | 23 |
| SC | SCF019 | Female | Subadult | 15 |
| SC | SCF020 | Female | Adult | 36 |
| SC | SCF021 | Female | Subadult | 26 |
| SC | SCF022 | Female | Subadult | 25 |
| SC | SCF023 | Female | Subadult | 24 |
| SC | SCF024 | Female | Adult | 24 |
| SC | SCF025 | Female | Subadult | 1 |
| SC | SCF026 | Female | Subadult | 15 |
| SC | SCJ001 | Female | Juvenile | 20 |
| SC | SCJ002 | Female | Juvenile | 16 |
| SC | SCJ003 | Female | Juvenile | 17 |
| SC | SCJ004 | Male | Juvenile | 11 |
| SC | SCJ005 | Male | Juvenile | 14 |
| SC | SCJ006 | Female | Juvenile | 10 |
| SC | SCJ007 | Male | Juvenile | 18 |
| SC | SCJ008 | Female | Juvenile | 20 |
| SC | SCJ010 | Male | Juvenile | 21 |
| SC | SCJ011 | Male | Juvenile | 19 |
| SC | SCJ012 | Female | Juvenile | 13 |
| SC | SCJ013 | Male | Juvenile | 12 |
| SC | SCJ014 | Male | Juvenile | 10 |
| SC | SCJ015 | Male | Juvenile | 6 |
| SC | SCJ016 | Female | Juvenile | 8 |
| SC | SCJ018 | Male | Juvenile | 8 |
| SC | SCJ019 | Female | Juvenile | 5 |
| SC | SCJ020 | Male | Juvenile | 7 |
| SC | SCJ022 | Female | Juvenile | 11 |
| SC | SCJ023 | Female | Juvenile | 3 |
| SC | SCJ024 | Female | Juvenile | 5 |
| SC | SCJ025 | Male | Juvenile | 1 |
| SC | SCJ026 | Female | Juvenile | 1 |
| SC | SCJ027 | Female | Juvenile | 1 |
| SC | SCJ028 | Female | Juvenile | 1 |
| LNNP | LNM001 | Male | Adult | 19 |
| LNNP | LNM002 | Male | Adult | 18 |
| LNNP | LNM003 | Male | Adult | 19 |
| LNNP | LNM004 | Male | Adult | 19 |
| LNNP | LNM005 | Male | Big bull | 17 |
| LNNP | LNM006 | Male | Big bull | 10 |
| LNNP | LNM007 | Male | Subadult | 19 |
| LNNP | LNM008 | Male | Subadult | 22 |
| LNNP | LNM010 | Male | Big bull | 26 |
| LNNP | LNM011 | Male | Adult | 23 |
| LNNP | LNM012 | Male | Adult | 17 |
| LNNP | LNM013 | Male | Adult | 23 |
| LNNP | LNM014 | Male | Adult | 24 |
| LNNP | LNM017 | Male | Adult | 19 |
| LNNP | LNM018 | Male | Adult | 22 |
| LNNP | LNM019 | Male | Big bull | 14 |
| LNNP | LNM021 | Male | Adult | 21 |
| LNNP | LNM022 | Male | Adult | 10 |
| LNNP | LNM023 | Male | Subadult | 14 |
| LNNP | LNM026 | Male | Big bull | 11 |
| LNNP | LNM027 | Male | Big bull | 14 |
| LNNP | LNM030 | Male | Subadult | 18 |
| LNNP | LNM031 | Male | Big bull | 20 |
| LNNP | LNM033 | Male | Subadult | 17 |
| LNNP | LNM034 | Male | Subadult | 15 |
| LNNP | LNM035 | Male | Subadult | 13 |
| LNNP | LNM036 | Male | Subadult | 14 |
| LNNP | LNM037 | Male | Adult | 20 |
| LNNP | LNM038 | Male | Adult | 12 |
| LNNP | LNM039 | Male | Adult | 24 |
| LNNP | LNM040 | Male | Subadult | 12 |
| LNNP | LNM044 | Male | Big bull | 13 |
| LNNP | LNM046 | Male | Adult | 21 |
| LNNP | LNM047 | Male | Subadult | 20 |
| LNNP | LNM049 | Male | Adult | 17 |
| LNNP | LNM050 | Male | Big bull | 6 |
| LNNP | LNM053 | Male | Big bull | 7 |
| LNNP | LNM054 | Male | Big bull | 1 |
| LNNP | LNM056 | Male | Adult | 15 |
| LNNP | LNM058 | Male | Adult | 8 |
| LNNP | LNF001 | Female | Adult | 21 |
| LNNP | LNF002 | Female | Adult | 22 |
| LNNP | LNF003 | Female | Adult | 21 |
| LNNP | LNF004 | Female | Subadult | 17 |
| LNNP | LNF005 | Female | Adult | 14 |
| LNNP | LNF006 | Female | Adult | 21 |
| LNNP | LNF007 | Female | Adult | 16 |
| LNNP | LNF008 | Female | Adult | 17 |
| LNNP | LNF010 | Female | Adult | 26 |
| LNNP | LNF011 | Female | Adult | 19 |
| LNNP | LNF012 | Female | Adult | 21 |
| LNNP | LNF013 | Female | Adult | 26 |
| LNNP | LNF014 | Female | Adult | 24 |
| LNNP | LNF015 | Female | Adult | 18 |
| LNNP | LNF016 | Female | Adult | 17 |
| LNNP | LNF017 | Female | Subadult | 14 |
| LNNP | LNF018 | Female | Adult | 14 |
| LNNP | LNF020 | Female | Adult | 25 |
| LNNP | LNF021 | Female | Adult | 25 |
| LNNP | LNF022 | Female | Adult | 13 |
| LNNP | LNF023 | Female | Adult | 14 |
| LNNP | LNF025 | Female | Adult | 16 |
| LNNP | LNF026 | Female | Adult | 21 |
| LNNP | LNF028 | Female | Subadult | 15 |
| LNNP | LNF029 | Female | Adult | 16 |
| LNNP | LNF030 | Female | Adult | 26 |
| LNNP | LNF031 | Female | Adult | 18 |
| LNNP | LNF035 | Female | Adult | 18 |
| LNNP | LNF037 | Female | Adult | 19 |
| LNNP | LNF039 | Female | Adult | 10 |
| LNNP | LNF040 | Female | Adult | 9 |
| LNNP | LNF042 | Female | Adult | 17 |
| LNNP | LNF044 | Female | Adult | 19 |
| LNNP | LNF045 | Female | Adult | 8 |
| LNNP | LNF046 | Female | Adult | 18 |
| LNNP | LNF052 | Female | Adult | 15 |
| LNNP | LNF054 | Female | Adult | 14 |
| LNNP | LNF055 | Female | Subadult | 13 |
| LNNP | LNF057 | Female | Adult | 14 |
| LNNP | LNF059 | Female | Adult | 9 |
| LNNP | LNF060 | Female | Adult | 9 |
| LNNP | LNF063 | Female | Adult | 15 |
| LNNP | LNF065 | Female | Adult | 20 |
| LNNP | LNF067 | Female | Adult | 19 |
| LNNP | LNJ001 | Unknown | Juvenile | 9 |
| LNNP | LNJ002 | Unknown | Juvenile | 5 |
| LNNP | LNJ003 | Unknown | Juvenile | 6 |
| LNNP | LNJ004 | Unknown | Juvenile | 8 |
| LNNP | LNJ006 | Unknown | Juvenile | 1 |
